# The Development of Anthropomorphism in Interaction: Intersubjectivity, Imagination, and Theory of Mind

**DOI:** 10.3389/fpsyg.2018.02136

**Published:** 2018-11-05

**Authors:** Gabriella Airenti

**Affiliations:** Department of Psychology, Center for Logic, Language, and Cognition, University of Turin, Turin, Italy

**Keywords:** anthropomorphism, development, pretense, intersubjectivity, theory of mind, imagination

## Abstract

Human beings frequently attribute anthropomorphic features, motivations and behaviors to animals, artifacts, and natural phenomena. Historically, many interpretations of this attitude have been provided within different disciplines. What most interpretations have in common is distinguishing children’s manifestations of this attitude, which are considered “natural,” from adults’ occurrences, which must be explained by resorting to particular circumstances. In this article, I argue that anthropomorphism is not grounded in specific belief systems but rather in interaction. In interaction, a non-human entity assumes a place that generally is attributed to a human interlocutor, which means that it is independent of the beliefs that people may have about the nature and features of the entities that are anthropomorphized. This perspective allows us to explain the problems that emerge if we consider anthropomorphism as a belief: (i) adults under certain circumstances may anthropomorphize entities even if they perfectly know that these entities have no mental life; (ii) according to the situation, the same entity may be anthropomorphized or treated as an object; (iii) there is no consistency among the entities that are anthropomorphized; (iv) there is individual variability in anthropomorphization, and this variability derives from affective states rather than from different degrees of knowledge about the entity that is anthropomorphized or greater or lesser naivety of the person who anthropomorphizes. From this perspective, anthropomorphism is a basic human attitude that begins in infants and persists throughout life. The difference between adults and children is not qualitative but rather a matter of complexity.

## Introduction

Human beings frequently attribute anthropomorphic features, motivations and behaviors to animals, artifacts, and natural phenomena. Historically, many interpretations of this attitude have been provided within different disciplines (see Guthrie, 1993 for an extensive treatment of various perspectives). What most interpretations have in common is that they distinguish children’s manifestations of this attitude, which are considered “natural,” from adults’ occurrences, which are considered exceptional and must be explained (Caporael and Heyes, 1997; Epley et al., 2007; Dacey, 2017). Particular circumstances, for instance, uncertainty, fear, helplessness would justify among adults the attribution of anthropomorphic characteristics to invisible and threatening causes of diseases, such as bacteria or viruses. Some particularly dangerous natural events, such as thunderstorms or fires, may also be described in anthropomorphic terms.

A notable exception to the idea, originally introduced in developmental psychology by Piaget (1926/1929), that animism is primarily children’s manifestation of irrational thinking, which is overtaken in adult life, is the position expressed by Guthrie (1993). Guthrie maintains that animism and anthropomorphism, far from being irrational, are reasonable answers to the ambiguity of the perceptual world. Guthrie proposes the following example. If you are jogging in a region that is well known for the presence of bears, at a first glance, you will most likely misinterpret boulders as bears. In fact, these momentary illusions show that people respond to perceptual ambiguity using the strategy of “better safe than sorry.” This strategy is dictated by the necessity to discover possible real threatening agents, and it is not specifically human but rather shared with other animals (Guthrie, 2002). According to this view, animism and anthropomorphism should be considered on a continuum. People interpret the world with humanlike models because human thought and action are the highest organization that they know. Religious anthropomorphism is then the “highest pitch” of a strategy of attributing to the external environment as much order and meaning as possible.

In the following, I shall argue that Piaget’s position claiming that children are particularly prone to anthropomorphism because they have not yet developed rational thinking is untenable. I shall also argue that Guthrie’s definition of anthropomorphism in terms of an adaptive form of perception does not account for the use of anthropomorphism in everyday life. I shall argue that anthropomorphism is a particular form of interaction with non-humans that children implement early in their development and that persists in adult life.

I will begin my argument by discussing the very concept of anthropomorphism.

## What Anthropomorphism Is and What It Is Not

Let us first define animism, anthropomorphism and their relation.

The term animism is generally used to refer to the attribution of intentional action and a general concept of “life” to objects and natural phenomena. Anthropomorphism is more specifically the attribution of human mental states or affects to non-humans. These two concepts are distinct and at the same time strictly connected. We could say that animism is a weaker form of anthropomorphism. However, when humans attribute life to non-humans, they often also attribute to them human mental and affective states.

To outline all of the forms that animism and anthropomorphism can take is a major task. Let us try, nevertheless, to propose some distinctions.

A first phenomenon that we could define as anthropomorphism is perceptual. This phenomenon is illustrated, for instance, by “seeing faces in the clouds,” to quote Guthrie (1993). Humans may identify perceptual characteristics of living beings in natural objects. For example, we can see a human face in the moon or a horse in the clouds. This form of imagination seems to be very basic in humans. We find fascinating examples of this in prehistoric caves, where sometimes we discover that the natural form of a wall has been underlined by a painter, who in this manner made it appear that s/he “saw” in it the outline of an animal. Humans frequently use fantasy to go beyond sheer facts and include simple objects or images in narrative contexts, which make them appear more appealing and meaningful. However, I doubt that phenomena of this type may be considered as a form of animism or anthropomorphism rather than a simple manifestation of human imagination. In fact, the perceptual aspect, the mere recognition of a human or animal form does not correspond to the definition of animism, even in its weaker form. After all, the recognition of human or animal features in a group of clouds is only one possibility among others. In clouds, we may see also artifacts, such as a coach, or other natural objects, such as a waterfall or a tree.

The process of imaginary transformation may become particularly salient in some cases when our fantasy is elicited by strong feelings. The fact that we can see a dangerous animal in a rock is not different from transforming an accidental noise behind us when we are walking on a dark and solitary street into the footsteps of a potential attacker. On other occasions, we can momentarily recognize in a stranger walking on the street someone that we long to see even if we know that it is not possible. In these situations, we materialize the objects of our fears or desires. However, these are brief illusions that quickly disappear.

As maintained by Guthrie (2002), there are reasons to think that these illusions are also present in the animal world. He proposes examples, some of them taken from von Uexküll (1934/1992). For instance, a starling was observed catching, capturing and finally swallowing a fly that was not there, a “magic” phenomenon according to Uexküll, and a product of imagination according to Guthrie. In this case, a strong “feeding tone” in the starling world would have “forced” the imaginary fly to appear in the absence of a real stimulus. Such a situation supports Guthrie’s point of view that there is no neat separation between humans and other animals when imagination is an almost instinctive response to the environment, dictated by the subject’s present world “tone,” to use Uexküll’s beautiful expression. However, here again we are not considering a case of animism. A phenomenon of this type is simply, at least in the human world, an unintended mistake. If a person is able to recover her or his cool head, the illusion disappears, and she or he immediately recognizes the misinterpretation.

Thus, contrary to Guthrie, I consider that anthropomorphism - also in its weaker form, i.e., animism – is not of a perceptual nature. Just seeing a human face in the moon is not an attribution of intentional life. What may transform our imagining the moon as a face from a simple fantasy into an anthropomorphic experience is the fact that we attribute an intentional stance to that face. We can imagine, for instance, that the moon looks back at us and that attitude could be defined as animistic. Anthropomorphism would appear, for instance, when, once this attribution of a simple intentional state is realized, we may start to think that the face shares our sadness or happiness or that it questions us, or we may even see it as menacing or foolishly indifferent to our feelings.

Following this approach, one may say that even in the case of threatening events such as a thunderstorm, fire, or disease, it is not the event itself that is anthropomorphized but rather the relation that a person establishes with it. A typical context that is suggested in these circumstances is a battle in which people feel engaged against the aggression of an evil force/intentionality that aims to destroy them or their assets. The language used is explicitly intentional, and this justifies an equally intentional response. For example, American firefighters “see forest fires as devious and as lying in wait,” and think that they must track them down (Guthrie, 1993). The personification of fatal diseases transforms the period of illness that a person painfully endures into a fight and death into the heroic fall in a battle. In a radio broadcast, a high-level athlete who had to interrupt her activity due to physical problems described her coming back to competition as the result of her managing making a deal with her body that was personified and observed as separate from her.

What we have said about natural facts or events is much more evident when we analyze the other possible objects of anthropomorphization, i.e., artifacts and animals. Regarding artifacts, we anthropomorphize those that “do” something for or with us. Not surprisingly, robots or computers are the mechanisms that we most anthropomorphize, as they are purposefully constructed to interact with humans (Airenti, 2015b).^1^ However, simpler devices that produce a useful activity, such as a coffee maker, a cash machine, or an alarm clock, are also supposed to “cooperate” with us. We may also anthropomorphize objects that we see as obstacles to our action, such as a door that does not open. We may even curse the door as if it intentionally resisted our attempts to open it. In fact, cooperation and hindering are connected, as we feel as an obstacle the fact that something that *should* cooperate with us actually does not. A door should be cooperative and let itself be opened. Thus, any object that can cooperate with us or hinder our activity may be the target of an anthropomorphic attitude.

Finally, humans may anthropomorphize animals. For animals, the process of anthropomorphization is more subtle because animals are living beings and do have cognitive capacities. The study of animal cognition, which assesses cognitive abilities across species and their similarities with humans, poses many methodological problems. However, it is largely accepted that animals have cognitive systems (Andrews, 2015). Most animals experience pain-like states (Bateson, 1991; Sneddon et al., 2014) and have at least basic emotions (Panksepp and Biven, 2012). Thus, the attribution of a mental life to animals is not completely due to anthropomorphism. However, the interesting point here is that the anthropomorphization of animals does not always occur, and it is often difficult to explain why the process of anthropomorphization is enacted in certain cases and not in others.

Eddy et al. (1993) found that a number of factors influenced human subjects’ attribution of cognitive abilities to animals, including perceived similarity of the animal to humans, its phylogenetic group membership and, in the case of dogs and cats, the degree to which they had formed an attachment bond with a particular animal. It seems natural that a higher level of anthropomorphization is triggered by pets, who are often considered companions with whom one can share her or his life. In fact, it has been shown that ownership of animals influences the reporting of emotions in animals, in particular secondary emotions (Morris et al., 2012). A study has shown that ownership of birds, rabbits, and rodents significantly increases the number of emotions that are attributed to those species (Wilkins et al., 2015). However, this study also showed that emotions are not consistently attributed even among mammals. The great majority of the participants also attributed secondary emotions to dogs. Only a few attributed them to cows. This result can be explained by the fact that in modern urban life, dogs are pets and cows are not. At the same time, participants also attributed emotions to animals that society either destroys as pests or keeps to use. Also, unexpectedly, Podberscek (2009) found that South Koreans might be in favor of keeping dogs as pets and at the same time against a ban on dog eating. On the other hand, most South Korean people were against both eating cats and keeping them as pets.

Thus, evidence shows that humans are rather incoherent in their attitudes toward animals. According to Serpell (2009), this incoherence is explained by humans’ desire to maintain the possibility of both having animals as companions and using them for their needs. To this aim, they “compartmentalize” and establish differences between animals, differentiating also the obligations that they have toward them. This disparity is supported by the fact that, as it has been shown, anthropomorphism is explained more by affection than by simple ownership. Increased attachment levels result in the increased use of emotive terms to describe animal behavior (Kiesler et al., 2007). Other studies have shown that owners attribute advanced human capabilities and emotions to their own animals but not to animals owned by others (Fidler et al., 1996) and that owner attachment influences the attribution of mirrored emotions to animals (Martens et al., 2016). Thus, it appears that it is our relation to the animals that influences our beliefs about their human-likeness and not the other way around.

This conclusion shows that even in the case of animals, which are living beings and thus most susceptible to being anthropomorphized, it is not the belief (for instance, regarding the existence of secondary emotions among them) that causes our attribution of human-like characteristics. The belief comes *a posteriori*, and it is often difficult to arrange it in a coherent and rational manner. It can also be noted that usually, transforming attitudes toward different animals into a coherent system of beliefs is not considered necessary. Inconsistencies are manifest only when researchers induce subjects to provide explicit judgments in experimental situations.

In the literature, the problem of anthropomorphism toward animals is particularly debated due to the moral issues that it involves.^2^ My purpose here is not to contribute to these debates. My aim is to outline the emergence and development of anthropomorphism to better comprehend how it manifests in different situations and toward different objects. The most salient fact that appears from the brief summary provided above is that humans may anthropomorphize almost any object, event, or animal. The characteristics of these entities are too disparate to provide an explanation for anthropomorphism. What do reproaching one’s car that does not start on an icy morning and accusing one’s cat of jealousy have in common? If the similarity is not in the entities that are the target of the process of anthropomorphization, we have to investigate the relational context in which anthropomorphism is activated. To pursue this aim, I will now analyze the beginning of anthropomorphism in young children.

## Children’s Animism in Piaget’s View

An analysis of animism in children was extensively performed by Piaget (1926/1929). He maintained that children have a spontaneous animist attitude that develops through different stages until around the age of 12. Piaget distinguishes two periods in children’s animism. The first, lasting until the ages of 4 and 5, is characterized by what he calls an integral and implicit animism. When a child adopts this attitude, “anything may be endowed with both purpose [*intention* in the original] and conscious activity according to the occasional effects on the child’s mind of such occurrences as a stone which refuses to be thrown on to a bank, a wall which can hurt the hand, etc.” (p. 213). In the successive period, implicit animism progressively disappears, and the process of systematization begins to follow discernable stages. It is in this period that it is possible to question the child. It must be noted that Piaget’s definition of animism includes anthropomorphism since in his examples children often attribute to entities of the world not only life and activity but also mental and affective states typical of human beings. Piaget writes, for instance, that “…the facts just stated show clearly enough the child’s *belief* [italics is mine] in animism and in an animism that is not very theoretical (its object is not to explain natural phenomena), but affective. The sun and moon take an interest in us (*ibid.*, p. 220).”

One important point is how Piaget obtained his data about children. He asked them questions about their beliefs. For instance, he asked, ”Does the sun move?” “Yes, when one walks, it follows. When one turns round it turns round too,” answered Jac, a 6-year-old. Most of the children he questioned, including some 11- to 12-year-olds, gave similar answers. To these answers, he responded with questions such as “If you and I were both walking but in opposite directions which of us would it follow?” Piaget was aware that this form of direct questioning, including drawing attention to resulting inconsistencies, made children express in the form of a belief something that they most likely had never thought about before. He put them in a position to search for responses to questions they would never had spontaneously posed to themselves. Therefore, they had to strive to find a solution to contradictions they did not imagine. However, the similarity of responses produced by children of the same age made him confident about the reliability of his results.

It is interesting to analyze the bases on which Piaget poses the distinction between the first and second periods of children’s animism. What does it mean that the first form of animism is implicit and integral in young children? For Piaget, at the beginning, children do not distinguish their own mental life from the external world. They think that everything in the world shares their own subjective life; between the self and the external world, there is *indissociation*. “Child animism presupposes a primitive state of belief in a continuum of consciousness” (*ibid.*, p. 231). Actually, children described all moving objects as conscious and every event as intentional. “The wall who hit me” said Nel, a 2.9-year-old girl who scratched herself against a wall, for instance. Natural objects are either good or naughty according to their activity; for instance, the rain may be naughty and the light nice. For a young child questioned by Piaget, the rain was naughty: “because Mummy pushes the pram and the pram all wet.”

Later, children develop a systematic animism, i.e., a set of explicit animistic beliefs. These beliefs are based on the principle of *introjection*. “All that either resists or obeys the self is thought to possess an activity as distinct as that of the self which commands or tries to overcome the resistance” *(ibid*., p. 242). The process of introjection derives from *egocentrism*, children’s characteristic self-centeredness. In this phase, when pushed to explain their animistic beliefs – for instance, that the sun follows them when they walk – children try to find reasons, to manage contradictions, etc.

In conclusion, animism, in Piaget’s view, is a step in the development of thought and is explained by the child’s egocentrism. Later, when children develop causal thinking, they free themselves from this form of irrational reasoning. From this same perspective, Piaget thinks that animism in adults is present only among “primitive” people. Members of such societies, according to him, are completely dominated by respect for tradition and do not develop the cooperation that in advanced societies allows children to overcome egocentrism. As a consequence, they never attain, even as adults, the stage of rational thinking (Piaget, 1928).

Many aspects of Piaget’s vision of development have been challenged. In particular, the fact that infants do not distinguish their internal life from the external world has been contested (Trevarthen, 1980; Stern, 1985/2000). However, Piaget’s point of view is still considered as the main reference regarding children’s animism, including his idea of animism as a form of irrational thinking that, in modern societies, disappears in adult age.

On this topic, let me provide a few remarks.

The most general point that we can contest is that animism is mainly a child’s (and a “primitive”) disposition. As we have observed in the previous section, adults practice many forms of anthropomorphism, and anthropomorphism is involved in most religious thinking in all societies. Thus, it is difficult to attribute it to confusion between the self and the other, to egocentrism, and, in general, to underdeveloped reasoning abilities.

Another point concerns the distinction made by Piaget between two forms of animism and attributed by him to different stages of development. The first manifestations of animism that Piaget detects in young children’s words are very similar to the situations in which adults resort to anthropomorphism. If it rains on a day when I planned gardening, I will most likely address the rain as if it were naughty and as if it intentionally hindered my activity. At the same time, Piaget introduces the principle of introjection, which connects animism to the idea of an object “obeying” or “resisting” the self. Actually, it is very difficult to detect in these interesting descriptions of children’s forms of animism, as Piaget would like, different steps of the development of rational thinking. The developmental path from indissociation to introjection is rather obscure, and it appears that there is no clear distinction between the first forms of animism and the manifestations of introjection that Piaget attributes to the phase of systematization. In all cases, Piaget refers to *beliefs* that children entertain. In fact, his questioning of children in the phase of systematization is mostly about the sun and the moon and children’s ideas that they act as intentional beings interested in humans’ life. These ideas are presented as *explicit beliefs* or at least as beliefs that become explicit when children must answer questions about them. I argue that the adoption of the concept of belief, both implicit and explicit, in these situations must be analyzed in more detail. Does the fact that a child says that the rain is naughty mean that s/he *believes* that the rain is an intentional being? We do not expect that this would be the case for an adult in the same circumstances. Are children’s ideas about the sun and the moon beliefs or rather fantasies? We can consider that children’s lack of knowledge about the physical reality might be replaced by fantasies. Moreover, the fact that things are different from what they appear to be is something that must be learned. For centuries, humans believed that the sun goes around the earth, and according of a survey performed by the American National Science Foundation in 2014 (reported by Time), one in four Americans questioned about this topic gave the incorrect answer.

Connected with what is presented above, there is a third question posed by Piaget himself. It concerns the role that language plays in children’s animistic expressions and what they take from adults’ discourse. Piaget concedes that adults often use finalistic language, producing, for instance, expressions like “the sun is trying to break through the mist” (*ibid.*, p. 248) However, in his view, language is not the cause of animism because this is the natural manner of children’s thinking. The similarity between adults and children would be only apparent because children take literally what for adults are only metaphors. Developmental research has shown that this is not the case, at least with respect to the distinction between physical and mental objects. Children by age 3 may use physical language to describe mental phenomena (as adult do), but they are aware of their different natures. A real object can be touched, whereas the thought or memory of the same object cannot be (Wellman, 1990). Thus also in the case of animism, we should be cautious to attribute a belief using mere linguistic evidence.

A final point regards an aspect missing from Piaget’s analysis. Actually, in his analysis of anthropomorphism, he never mentions pretense. He considers animism as an underdeveloped form of thinking, and he does not contemplate the connection that it might have with the world, so important for children, of pretense and fantasy. In pretend play, children attribute at least animacy, but often also mental and affective states, to puppets, dolls, stuffed animals, fictional characters, and even simpler objects, such as blocks or pebbles. The fact that children at 18 months start to deal with narrative and fantasy situations in which intentionality and other mental and affective states are attributed to non-humans is possibly connected to other forms of animism that children perform. Moreover, young children are often involved in relations with house pets that they consider as companions and with whom they play. It must also be stressed that these forms of animism are often favored by adults who consider them suitable for children.

In conclusion, are we confronted with different forms of anthropomorphism (implicit and explicit, for instance) in the cognitive development of the child? Do we have to appreciate the role played by language? Is there a relationship with pretend play? To provide an adequate account of anthropomorphism, we should consider all of these aspects, which will allow us to distance ourselves from the too-simple vision that animism can be reduced to children’s naive beliefs about entities of the world. Actually, anthropomorphism is a much more pervasive attitude that starts early and persists in different manners throughout life. Moreover, it plays an important part in the interactions between children and adults.

## The Development of Anthropomorphic Thinking: From Objects in Motion to Pretense

The tendency to interpret in human terms very simple objects in motion has been demonstrated in a long experimental tradition since the seminal work of Heider and Simmel (1944). They showed subjects a brief film in which three geometrical figures – a large triangle, a small triangle, and a circle – appeared, moving in different directions and at different speeds. The only other figure in the field was a rectangle, a section of which could be opened and closed. When asked to describe the scene, most subjects interpreted the movements of the geometrical figures as the actions of human beings and as part of a connected story. These results were replicated with adults (Oatley and Yuill, 1985) and children (Berry and Springer, 1993; Springer et al., 1996), and what is particularly interesting is that young children succeeded in adapted versions of this experimental paradigm. Montgomery and Montgomery (1999) showed that by the age of 3, children inferred goals from the movement of balls and distinguished goals from the outcomes of the acts. Gergely et al. (1995) showed that 12-month-old children expected that colored dots on a screen pursued their goals as an intentional actor would do and were surprised if this was not the case.

Researchers have tried to identify the visual cues that produce the effect of animacy and to elucidate the relation between perception and higher-level forms of inference (Dasser et al., 1989; Scholl and Tremoulet, 2000; Scholl and Gao, 2013; van Buren et al., 2016). However, for the present argument, the point is that when seeing forms in coherent motion, humans since a very young age naturally attribute to them intentionality and reciprocal interactions; for instance, they think that a figure is chasing another or tries to join it.

Along the same lines are the results of experiments regarding the development of sociomoral evaluation in infants. In this experimental paradigm, infants viewed a colored wooden block with eyes attempting to achieve a goal, i.e., climb a hill. The attempt could be facilitated or hindered by another block, who pushed the protagonist up or down the hill. By 3 months, infants looked longer at individuals who facilitated the protagonist’s goal than at those that blocked its goal (Hamlin et al., 2007, 2010). This experimental paradigm in all its variations has allowed for the formulation of very interesting hypotheses about intuitive morality in infants (Wynn and Bloom, 2013; Van de Vondervoort and Hamlin, 2016).^3^ Regarding anthropomorphism, one aspect is particularly relevant. The evaluations are made possible by the fact that infants naturally attribute good or evil intentions to geometrical objects moving on a screen. Let us focus on the developmental path. If we compare the interpretations of the movements of simple objects made by adults with those made by children, the difference between them seems to be only in terms of complexity. As Heider and Simmel (1944) show, adults may imagine complex stories involving the “characters,” whereas the younger the children, the simpler the reaction. In infants, we can register only surprise if the “actors” do not coherently pursue their supposed goals or a preference for cooperative behavior over a hindering one. However, the anthropomorphic attribution is present in both groups. When objects move in a coherent manner with respect to one another, they are not only interpreted as causally linked (Michotte, 1946/1963) but also as interacting.

A particularly interesting point is that the language used to describe these situations is affected. As we have observed in the studies with infants mentioned above, the researchers themselves describe the experimental situation using anthropomorphic language, a block *pushing* the other up or down. Actually, describing the situation in purely geometrical objective terms would be difficult, long, and barely comprehensible, as Heider and Simmel write in the *Methods* section of their paper: “A few ‘anthropomorphic’ words are used since a description in purely geometrical terms would be too complicated and too difficult to understand” (p. 245). Thus, not only the experimental subjects but also the authors of the studies and the readers are involved in anthropomorphic attribution. We find exceptions to anthropomorphic interpretation of objects in motion only in clinical groups, such as persons with autism spectrum disorders (Abell et al., 2000; Klin, 2000).

A fundamental feature of anthropomorphism that appears already in infancy is the fact that in these interactions, two possible roles are attributed to the actors. One character may either cooperate with or be an obstacle to the other’s supposed goals (Tomasello and Vaish, 2013). According to the age of the subjects, this simple dichotomic distinction may appear at different levels of elaboration, but it is still present in adult anthropomorphization of objects. As said before, in everyday life, we expect that objects *cooperate* with us to ensure the success of our activities. In general, this “collaboration” is not an issue (people do not wonder about their coffee maker’s intention to produce coffee), but when some event compels them to focus on their relation with the object, such as when they are unsure about how to proceed or fail to reach their goal, the object enters the focus of attention and may be anthropomorphized. One can address it and invite it to be more collaborative or blame it as an obstacle to achieving the intended goal, for example.

The analysis of the geometrical objects in motion may be pursued further. The original experiment showed that adults were very easily induced to connect the simple acts performed by the figures and construct stories. This observation means that even the simplest situations may trigger the process of imagination. The geometrical figures are not only perceived as acting in a manner related to each other but also attributed mental and affective states. For instance, in Heider and Simmel’s experiment, two triangles were described by adults as two men fighting for a girl (represented by a circle). In this case, the adults were exercising an ability that begins with children as young as 12 months in pretend play (Fein, 1981).

Pretense in children involves both anthropomorphization and imagination. Young children may naturally produce situations similar to the ones proposed in the experiments mentioned before, for instance, using colored blocks to represent objects and imagine simple stories involving them. They anthropomorphize and construct stories with stuffed animals, puppets, and dolls. However, even when young children anthropomorphize the objects with which they play, they are not confused about their status. It has been shown that at least by age 3, children distinguish reality from pretense (Woolley and Wellman, 1990; Harris, 2000; Ma and Lillard, 2006) and that differences between children and adults reflect a continuous development (Woolley, 1997). Moreover, children’s creation of imaginary worlds is often a social construction (Leslie, 2002) that involves adults. Already when they are 15-month-old, children engage with mothers in reciprocal imitation of pretense actions, and mothers’ imitation predicts children’s pretending (Markova and Legerstee, 2015).

The role of adults in leading children to anthropomorphism clearly appears in children’s storybooks, cartoons, and movies, which often contain anthropomorphized animals and objects. The use of anthropomorphization of animals for children has been recently questioned in the literature, and a number of studies have shown that it does not necessarily enhance early learning (Richert et al., 2009; Ganea et al., 2014; Geerdts, 2016). From a theoretical point of view, the question is whether anthropomorphism is a natural form of thinking typical of young children that evolves in later years, as maintained by Carey (1985), or instead if it develops under the influence of adults and the cultural milieu. In this debate, the term anthropomorphism is often replaced by anthropocentrism to stress the fact that using human categories to understand other biological entities leads to mistaken representations. According to Carey, young children reason about animals from an anthropocentric point of view that is later abandoned due to a conceptual change. In contrast with this view, interesting results show that anthropomorphism in young children’s dealing with biological entities is not universal. It seems to be absent, for instance, in rural cultures (Medin et al., 2010). Additionally, in urban cultures, it is not present at 3 years of age but rather develops later (Herrmann et al., 2010). What these studies show is that there is not a universal developmental stage that involves the extension of anthropomorphic features to unknown biological entities. Anthropomorphism is an attitude that children acquire in urban societies in which animals are not part of everyday life except as pets and companions.

The evidence presented in this section leads us to some conclusions about the human tendency for anthropomorphism. There are aspects of anthropomorphism that seem to be universal and that emerge very early in development. Let us summarize them.

(1) Humans rarely, if ever, interpret coherent movement of multiple entities without resorting to anthropomorphism, and this is true both for adults and for children since infancy. As we have observed, adults have no vocabulary other than anthropomorphic terms for these situations. The description in geometrical objective terms of what we call a block “pushing” another is difficult to produce and even more difficult to understand. This is more than a linguistic problem. Intentionality is the best model that humans have to describe these situations.
(2) The above observation means that causal thought is insufficient to explain these facts and that the entities are conceived as related and in interaction. One entity is perceived as trying to join or escape another, for instance. Thus, another anthropomorphic concept seems to be unavoidable, *relation*. Entities in a defined space that move in a coherent manner are related to one another as if they were human beings.
(3) A relation of this type has two basic forms of expression, cooperation, and competition. One entity may collaborate with another or be perceived as an obstacle. Again, this is true for children and for adults. Objects are perceived as helpers or hinderers. Thus, even in the simplest relational contexts, we do not find animism but rather anthropomorphism. Note that there is nothing in the object itself that makes it adapted to be anthropomorphized, nor is there any particular belief leading to anthropomorphic attribution of mentality. Anthropomorphism is grounded in the relation.
(4) Establishing these basic forms of relation implies evaluation. Infants already distinguish the two situations and exhibit a preference for the cooperative object over the non-cooperative one. The whole process is made possible by imagination. Objects acquire imaginary characteristics, including mental and affective states, and more complex relations may be evoked. This process starts in young children but is still present in adults even if the imaginary constructions may be differently elaborated in the two cases.

We can conclude that in humans from infancy to adulthood, there is a basic tendency to anthropomorphize entities under certain circumstances, i.e., that an entity be perceived as in a human-like relation with them.

It is important to stress that this attitude certainly appears in infancy but is present throughout life. Anthropomorphism is a specific human attitude, not a childish mistake. In this respect, separating young children’s attitudes from adults’ is unsuitable because it hides the fact that children construct their anthropomorphic attitudes in interactions with adults who not only normally use an anthropomorphic language but also share pretend play with children and propose to them entertainment in which anthropomorphism is dominant.

However, what about the anthropomorphization of animals? As we have observed, the experimental results do not confirm that it is universal in young children. On the contrary, it is acquired specifically in societies in which contact with animals is not frequent. As stressed by Herrmann et al. (2010), if we induce young children to categorize, they do so according to animacy, i.e., following the distinction between animate objects and inanimate objects, a distinction that young children already make in the first year of age (DeLoache et al., 2011). ^4^ This is consistent with experiments showing that children at 6 months of age exhibit a preference for natural situations in which an experimenter speaks with a person or grasps an object relative to unnatural situations in which the experimenter grasps a person or speaks to an object (Molina et al., 2004).

According to this perspective, the basic distinction that young children make is between animate and inanimate entities. On the contrary, attribution of specifically human features to animals would be acquired. Children who have no information about animals are taught to use the human model to interpret their behavior. Reciprocally, anthropomorphized animals are used to teach them behavioral and moral rules of human society. In societies in which animals coexist with humans, children better know about them and have more specific models to interpret their behavior. What is important here is that this claim applies to beliefs about animals and must be distinguished from interaction with them. When interacting with animals, children who have pets may treat them as companions and anthropomorphize them as adults do.

The previous remarks illustrate a fundamental distinction between anthropomorphism as a belief and anthropomorphism as it appears in interaction. In my perspective, to treat anthropomorphism as a system of beliefs without considering its relational aspect is a source of misunderstanding and potentially contradictory results. In the following, I shall argue this point in more detail.

## Anthropomorphism in Interaction

As we have observed, when anthropomorphism is defined as a system of beliefs, a distinction between strong and weak beliefs is often accepted. Beliefs may be strong, such as anthropomorphic traits attributed to God in many religions, or weak, as in the case of mental states momentarily attributed to objects such as a car or a computer. For instance, in their theory of anthropomorphism, Epley et al. (2007) maintain that the weaker forms are better described as “as if metaphorical reasoning.” However, they conclude, “the difference between weak and strong versions of anthropomorphism, we suggest, is simply a matter of degree regarding the strength and behavioral consequences of a belief, not a fundamental difference in kind (p. 867).”

Let us examine why it is not useful to characterize anthropomorphism as a form of belief.

Let us consider the concept of a “strong belief” as part of an anthropomorphic system of beliefs. As we have discussed in Section “What Anthropomorphism is and What it is not,” any entity can be anthropomorphized, including artifacts and biological entities such as plants and animals. People may anthropomorphize not only cats and dogs but also pests, robots or locks. There is no requirement of human-likeness or a high level of complexity. Moreover, the same entity may be treated by the same person alternately in both anthropomorphic and realistic manners, showing that this attitude is independent of the knowledge about the entity that one possesses. The uncertainty that a person may entertain about the real nature of an entity is not an explanation either. Obviously, anybody knows the fact that a mammal is much more similar to a human being than an insect, and people are more likely to attribute complex cognitive states to primates than to cockroaches (Eddy et al., 1993). Thus, people have a more or less conscious concept of *scala naturae.* However, under certain circumstances, an insect can also be anthropomorphized. Inversely, a cow may be objectified when it is used as food.

Let us look now at the idea of “weak belief.” As we have observed, the metaphor model is presented by some authors as a weaker form of belief, a belief that has a reduced behavioral impact. Can the anthropomorphizing process be considered a form of metaphor? Actually, the metaphor model is too generic to explain the process of dealing with non-humans as if they were humans. Furthermore, the concept of metaphor is inadequate in this context because the aim of anthropomorphic process is not to describe a situation but rather to affect it. We have repeatedly observed that in anthropomorphic representations, the content is irrelevant. Only the relational context transforms a representation into an instance of anthropomorphism. The activation of the process of anthropomorphization of an object momentarily obscures the realistic knowledge about it that one has. However, the situation is easily reversed, and the object can be perceived again with its actual features. In all of the cases that we have observed, anthropomorphization is never a question of degrees. It is an all or nothing attribution, a figure–ground relation.

My hypothesis is that to explain the existence of inconsistent points of view about the same object, we have to define the circumstances in which this shift from one point of view to the other occurs. Anthropomorphism is neither a belief in its stronger forms nor a metaphor in its weaker forms. Fundamentally, anthropomorphism is a way of relating with a non-human entity by addressing it as it were a human partner in a communicative situation.^5^

Anthropomorphizing objects or biological entities is a means to establish a relation with them, dealing with them as interlocutors in a communicative interaction. This process leads to the *automatic* attribution of intentionality and social behavior. The anthropomorphic relation has two basic modalities, cooperation and competition. When I establish this type of relationship, I expect that the entity cooperates to the achievement of my goals, and I use communicative means to urge cooperation. In case I perceive it as an obstacle, I fight to overcome it. Obviously, all of that is imaginary. My car will not become more efficient because I speak to it, and unfortunately, my chances to win a lottery will not increase because I implore fate to help me. The crucial point here is that no belief, weak or strong, is involved in this situation, simply because people do not believe that cars or lotteries have human minds.

The most natural means for humans to influence others’ actions and to gain their cooperation is to communicate with them, and this implies the attribution of mental and affective states. This same modality is employed with non-human entities in the process of anthropomorphization. Thus, one can speak to, complain, scold, justify, compliment, etc. any entity that he or she intends to address. The motivations may be multiple, such as uncertainty, fear, desire, hope, etc., but the format is the only one that humans know how to use to influence others, i.e., enacting a communicative interaction. In the case of the establishment of an anthropomorphic relationship, it will be an imaginary one.

This model is compatible with the evidence that there are individual differences in anthropomorphism (Waytz et al., 2010). Some individuals who lack social connections and feel lonely may be more disposed to establish imaginary relations with non-human entities. In the same manner, a sick person may feel less weak and helpless if s/he consider his or her illness as an enemy to fight.

This approach allows us to see from a different perspective the comparison between adults and children with respect to anthropomorphism. The most accepted position maintains that there is variability among adults but that a fundamental difference exists between adults and children. Children would be more prone than adults to anthropomorphism (Epley et al., 2007). However, evidence shows that in both adults and children, anthropomorphism exhibits the same features.

We have defined anthropomorphism as a relation that a human establishes with a non-human entity. Such a relation is enacted by putting a non-human entity in the position of interlocutor in an imaginary communicative situation. Certainly, children are very soon acquainted with this format. On one hand, children participate in communicative interactions very precociously, well before language acquisition (Bateson, 1975; Bruner, 1975; Trevarthen, 1998; Liszkowski et al., 2012; Airenti, 2017). On the other hand, equally precociously, they learn to extend the communicative format to non-humans in pretense (Harris, 2000). We could even state that pretend play is the prototypical anthropomorphic communicative situation.

Children acquire the communicative format in interactions with adults, and in interaction with adults, they acquire the possibility to extend it to objects and biological entities, real or imaginary. Note that in their first interactions with infants, adults include them in communicative games in which children participate with simple sounds and smirks and adults with their much more complex gestural and verbal communicative repertoire. In these proto-dialogues, infants’ behaviors are interpreted (and sometimes overinterpreted) as intentional responses (Newson, 1979). Adults attribute to them mental and affective states that they do not necessarily experience. Thus, adults, at least in our society, often anthropomorphize infants. At the same time, they anthropomorphize animals, real or represented, and use them to teach children different aspects of mental, social life, and moral rules. Thus, if children have an attitude toward anthropomorphization, adults are equally prone to anthropomorphization when they relate to infants. More precisely, parent–child communication often involves a non-human as a third partner. Think of an example of this type. A mother indicating the child’s teddy bear tells her, “Look, he stares at you. He also wants you to drink your milk!” or “If you are not drinking your milk, he will.”

In both children and adults, what may change is the stability of the relation that is the basis of this process. In some cases, the relation is steady. This is the situation for the relation that a young child has with his or her object of attachment (a teddy bear, soft doll, piece of cloth, blanket, pillow, etc.). Just like adults, children do not attribute mental states to objects on the basis of perceptual similarity to living beings. In one study, children 3 years of age attributed significantly more mental states to their attachment toy than to their favorite toy (Gjersoe et al., 2015). For older children and adults in general, this is the relation that is established with a pet.

In other situations, the relation is momentarily established due to specific circumstances. In this case, the range of possibility is wide. Children, sometimes together with adults, engage in pretend play involving real or imaginary objects and animals. Children and adults anthropomorphize any type of object that may be invited to be more cooperative or blamed for a misdeed, for example.

The model is the same both in the cases of steady relations and temporary ones. The application of the communicative format implies that in both cases, (1) the actor perceives the interlocutor as intentional and (2) the interlocutor’s actions are perceived as addressed to the actor (Airenti et al., 1993).

Importantly, this model distinguishes beliefs from anthropomorphic attribution. The anthropomorphic attribution is independent of the possibility that humans entertain anthropomorphic beliefs about animals. This communicative format can always be suspended, and this shows that the anthropomorphic attribution is not based on beliefs. A child may discard without qualms a toy that she previously addressed as a partner in a fantasy game. An adult will drive her or his car without thinking that s/he has previously invited it to behave.

From this perspective, we can reconsider Piaget’s point of view regarding young children’s animism. According to him, children attribute consciousness and agency to all the entities of the world because they are not able to distinguish their own self from the outside world. Anthropomorphism is then a product of confusion, indissociation in Piaget’s terms, and is destined to disappear in adulthood.

In fact, if we adopt the interaction model that we have proposed here to explain anthropomorphism, it clearly appears that young children and adults collocate on a continuum. Young children, just like adults, manifest a human predisposition to involve in a communicative format non-human entities, and their attitude toward anthropomorphism is independent of their beliefs, whether they are true or false.

This perspective better explains the fact that cases of anthropomorphism that are taken as examples of children’s confusion are also very common in adults, such as accusing a wall of hurting or blaming the rain because it hinders a planned activity. Importantly, in this view, the first and second phases of children’s animism according to Piaget also appear in clear continuity. The second phase is characterized according to Piaget by the process of introjection, defined by him as “the tendency to situate in others or in things the reciprocal feelings to those we experience from their contact” (*ibid.*, p. 242). An illustration of this type of anthropomorphism is the fact that consciousness of pain presupposes the attribution of malice to the object that is source of it. This definition seems incongruous and difficult to explain if we consider that the attribution is the product of a belief. If we consider it from a relational point of view, it becomes very easily understandable. Reciprocity, in fact, is a basic feature of interactions (Airenti, 2010). Interlocutors expect that there is a reciprocal relation between their actions. Thus, one possible human means to react to a fact caused by a non-human is to personify the non-human and put it in the position of addressee in an interaction. This is not simply animism but rather anthropomorphism because in this case, attributing the role of interlocutor to a non-human entity implies the ascription of mental and affective states. If someone hurts her finger and blames the cause of it, it is the same if it is a door that closed unexpectedly or a pup’s biting. Beliefs about the intentionality of doors and pups are not in question. It is the position in a relation that implies the attribution. Thus, young children, older children and adults may have different beliefs about non-human entities, but in these situations, they react in a similar manner. At the same time, under different circumstances, young children, like adults, may behave toward the same non-human entities in a non-anthropomorphic, realistic manner.

## Conclusion

In this article, I have discussed the cognitive processes underlying anthropomorphism.

Some authors have proposed that the attribution of human mental states and emotions to non-human entities is based on the same brain mechanisms that humans have developed to understand other humans (see Urquiza-Haas and Kotrschal, 2015, for a review). All stimuli indicating animacy would automatically activate the social network in the brain. This process, according to Urquiza-Haas and Kotrschal (2015), combines with domain-general mechanisms such as inductive and causal reasoning more influenced by cultural differences and individual variability.

My hypothesis is that a crucial distinction has to be drawn between anthropomorphic beliefs and anthropomorphic interactions. The major tenet of my argument is that anthropomorphism is not grounded in specific belief systems but rather in a specific modality of interaction. In interaction, a non-human entity takes the place that is generally attributed to a human interlocutor.^6^ This process means that anthropomorphism is independent of the beliefs that people may have about the nature and features of the entities that are anthropomorphized. This perspective allows us to explain problems that emerge if we consider anthropomorphism as a belief: (i) adults under certain circumstances may anthropomorphize entities even if they perfectly know that they have no mental life; (ii) according to the situation, the same entity may be anthropomorphized or treated as an object; (iii) there is no consistency among the entities that are anthropomorphized; and (iv) there is individual variability in anthropomorphization, and the variability derives from affective states rather than from different degrees of knowledge about the entity that is anthropomorphized or greater or lesser naivety of the person who anthropomorphizes.

In the process of anthropomorphization, an imaginary dialogue is established with an entity. This format implies the attribution of mental and affective states. I argue that this format is the basis of any form of anthropomorphism. This format is activated any time a human relates with a non-human entity. What may change are the motivations that induce a human to establish a relation with an object, an event or a biological entity; the type of relation; and the complexity of mentality that is attributed. It is at this level that cultural differences are relevant. For instance, this process may influence the relationship that is normally accepted with animals. In Europe or in the United States, cats are typical house pets and are considered ideal companions, whereas in Korea, they are not accepted in this role. There is also space for individual variability. Even in a society that appreciates the value of the companionship offered by pets, the strength of the bond that individuals establish with them varies and with this the complexity of the attributed mentality, such as the attribution of secondary emotions, also varies.

From this perspective, it is also easier to understand anthropomorphism in children. Children very precociously acquire the communicative format that allows for anthropomorphization. Thus, they may apply it in the same manner that adults do. In this sense, there is no difference from adults. There is no reason to postulate a specific animistic form of thinking that would characterize only children and for which there is no evidence.

If we separate the activation of anthropomorphic attribution from the beliefs about non-human entities, the obvious fact that children’s knowledge about these entities is not as developed as adults’ knowledge is irrelevant. In fact, what appears when we question children about their beliefs is their limited knowledge and not an underdeveloped form of thinking. Possible differences only concern those aspects that affect variability among adults, i.e., the motivations, types of relations, and mentality attributed to non-human entities. These aspects are age-related. In particular this is true for the attribution of mental and affective states. In anthropomorphic attribution, children use the same theory of mind abilities that they use in interactions with humans and that correspond to their stages of development.

In conclusion, precociously acquired communicative and imaginative abilities will enable even young children to extend to non-humans the interaction format that they use in their everyday relations. Regarding the attribution of mentality, its complexity will depend on the current development of the theory of mind (Airenti, 2015a, 2016).

This approach is also useful to explain how adults and children influence each other in the anthropomorphic process that develops in their interactions. Though human predisposition toward anthropomorphism already manifests in infants, its use is so present in children because it is strongly supported by adults. Adults who are normally scarcely aware of their own use of anthropomorphism explicitly use it in their interactions with young children. They both encourage pretend play and storytelling in which non-humans – including not only animals but also other biological entities such as plants or objects – are anthropomorphized. The intent is often explicitly pedagogical. In this manner, children are supposed to acquire knowledge and social and moral rules. The underlying idea is that learning through, for instance, animal stories should be more natural and simpler for children. Actually, this belief is contradicted by experimental research. A number of studies have shown that children enjoy listening to stories but that learning is not favored by the presence of anthropomorphic characters. In fact, children are more likely to transfer to the real world knowledge derived from realistic stories than that from anthropomorphic stories (Larsen et al., 2018). Thus, the fact that anthropomorphism is a fundamental tool for children’s learning appears to be an adult bias. This topic is still understudied: clarifying adults’ vision of children’s anthropomorphism would be very useful to better understand anthropomorphism in general. Intuitively, one could say that adults anthropomorphize infants in the same manner that they do pets. When adults interact with infants, they attribute to them a theory of mind as complex as theirs. At the same time, adults constantly lead children toward anthropomorphism. All of these matters should be further explored. What is certain is that adults’ and children’s anthropomorphism are intertwined and that it is not possible to discuss children’s anthropomorphism without considering adults’ folk psychology about children.

In conclusion, in this article, I argued that anthropomorphism is not a form of belief but rather a means to establish a relation with non-humans as if they were human beings. Anthropomorphism is a basic human attitude that begins in infants and persists throughout life. The difference between adults and children is a matter of the growing complexity of the same mental processes.

## Author Contributions

The author confirms being the sole contributor of this work and has approved it for publication.

## Conflict of Interest Statement

The author declares that the research was conducted in the absence of any commercial or financial relationships that could be construed as a potential conflict of interest.

## Footnotes

1 The problems created by perceptive similarity between robots and humans have been first exposed by Mori’s work on the uncanny valley (Mori, 1970). Mori maintained that similarity to humans does not necessarily produce familiarity. In a graph considering familiarity as a function of robot’s appearance, as robots appear more human-like, humans’ sense of familiarity increases until a point where it plunges into the uncanny valley. Moore (2012) proposed a mathematical explanation of this effect. Gray and Wegner (2012) suggested that people may find robots “unnerving” because their appearance prompts attributions of mind.

2 One much-debated question concerns the cognitive and affective abilities that different species actually possess. This problem is connected with questions concerning animal rights and human obligations to promote their wellbeing. Another question is whether attributing human-like characteristics to animals is useful for understanding their nature and needs (Root-Bernstein et al., 2013).

3 In the literature, there has been much debate concerning the replicability and robustness of findings obtained within this experimental paradigm (Hamlin et al., 2012a,b; Scarf et al., 2012a,b; Cowell and Decety, 2015; Hamlin, 2015; Salvadori et al., 2015; Nighbor et al., 2017). Surely more research will be necessary to define the concept of core morality. For my argument, the fact that infants attribute intentions to geometrical objects (a fact that is largely recognized by researchers adopting different approaches) is sufficient.

4 Simion et al. (2008) have shown that discrimination between biological and non-biological motion and preference for biological motion is already present in 2-day-old babies.

5 I do not consider here anthropomorphism as it can be found in written or oral religious texts. In that case, we do have an explicit system of beliefs that people who adhere to one religion are supposed to share. However, these systems are built on anthropomorphic relationships (Severi, 2018).

6 A relation between anthropomorphism and communication has been proposed by Horowitz and Bekoff (2007) who suggest that anthropomorphization could occur when animals’ behavior follows the rules of human communication.
